# Complete Chloroplast Genomes of *Saussurea katochaete*, *Saussurea superba*, and *Saussurea stella*: Genome Structures and Comparative and Phylogenetic Analyses

**DOI:** 10.3390/genes14112002

**Published:** 2023-10-26

**Authors:** Hui He, Tao Wang, Chuyu Tang, Zhengfei Cao, Xiaojian Pu, Yuling Li, Xiuzhang Li

**Affiliations:** 1 Qinghai Academy of Animal and Veterinary Science, Xining 810016, China; he15226330573@163.com (H.H.); 13085500761@163.com (T.W.); chuyutang0410@163.com (C.T.); c1474477969@163.com (Z.C.); puxj@qhu.edu.cn (X.P.); 2 State Key Laboratory of Plateau Ecology and Agriculture, Qinghai Academy of Animal and Veterinary Sciences, Qinghai University, Xining 810016, China

**Keywords:** *Saussurea*, chloroplast genome, phylogenetic analysis, sequence variation, molecular markers

## Abstract

*Saussurea* plants are widely distributed in Asia and Europe; however, their complex phylogenetic relationships have led to many difficulties in phylogenetic studies and interspecific identification. In this study, we assembled, annotated, and analyzed the chloroplast genomes of three *Saussurea* plants: *Saussurea katochaete*, *Saussurea superba*, and *Saussurea stella*. The results showed that the full-length sequences of the three *Saussurea* plants were 152,561 bp, 151,452 bp, and 152,293 bp, respectively, which represent the typical quadripartite structure, and the genomes were relatively conserved. The gene annotation results showed that the chloroplast genomes of *S. katochaete*, *S. superba*, and *S. stella* were annotated with 128, 124, and 127 unique genes, respectively, which included 83, 80, and 83 protein-coding genes (PCGs), respectively, 37, 36, and 36 tRNA genes, respectively, and 8 rRNA genes. Moreover, 46, 45, and 43 SSR loci, respectively, and nine highly variable regions (*rpl32-trnL-UAG*, *rpl32*, *ndhF-rpl32*, *ycf1*, *trnC-GCA-petN*, *trnC-GCA*, *rpcL*, *psbE-petL*, and *rpl16-trnG-UUG*) were identified and could be used as potential molecular markers for population identification and phylogenetic study of *Saussurea* plants. Phylogenetic analyses strongly support the sisterhood of *S. katochaete* with *S. superba* and *S. stella*, and are all clustered with *S. depsagensis*, *S. inversa*, *S. medusa*, and *S. gossipihora*, of which *S. gossipiphora* is most closely related. Additionally, the phylogenetic results indicate a high frequency of differentiation among different species of *Saussurea* plants, and many different species or genera are morphologically very different from each other, which may be related to certain genetic material in the chloroplasts. This study provides an important reference for the identification of *Saussurea* plants and studies their evolution and phylogenetics.

## 1. Introduction

*Saussurea* originated in the early-middle Miocene within the Hengduan Mountains [1], and is an annual, biennial, or perennial herb of the Trib. Cynarea of the Compositae. There are more than 400 species worldwide, mainly in Asia and Europe, with about 264 species (66%) found in China, mainly in areas at altitudes of 400–5000 m [2]. Due to the wide distribution of the genus, the variety of species, and the unclear morphological differentiation between species, the identification of species is difficult [3,4].

More than 10 species of this genus have been used to treat bruises and sprains, altitude sickness, and food poisoning. In the last two decades, it has been found that the chemical constituents of the *Saussurea* plant mainly include compounds such as steroids, phenylpropanoids, flavonoids, sesquiterpenoids, and triterpenoids, which are known to possess biological activities such as antitumor, antibacterial, anti-inflammatory, and cardiotonic activities [5,6]. Because of its high medicinal value and because wild resources are not yet available for large-scale application, artificial cultivation is one of the future development trends. However, *Saussurea* plants are distributed in harsh environments, coupled with their controversial population identification [7] making accurate species identification one of the prerequisites for achieving cultivation.

Previous studies have shown that the plant chloroplast genome, which together with the central nuclear genome and the mitochondrial genome constitutes a complete plant cell, is an effective way to achieve species identification [8,9]. In addition, chloroplasts, like the mitochondria, are double-membrane-bound cytoplasmic organelles whose origin can be traced back at least one billion years to the prokaryotic organisms of a photosynthetic cyanobacterium [9,10,11]. Over a long period of evolution, chloroplasts have become indispensable organelles to allow green plants to carry out life activities such as photosynthesis and carbon fixation [12]. They are involved in the synthesis of fatty acids and amino acids in the plant body [13], and they possess a set of structurally complete genomes [14], which usually does not exceed 160 kb, including a large single-copy region, a small single-copy region, and two inverted repeat regions, [15]. Compared with the nuclear and mitochondrial genomes, they have the advantages of easy discernment of their age characteristics, easy access to sequences, a more conserved gene composition and structure, rich genetic information, and high species discrimination [9,16]. These characteristics are of great significance for the identification of *Saussurea* plant species, which have complex germplasm relationships and a rich diversity.

*S. katochaete* and *S. stella* are both perennial stemless rosette-like herbs. In China, they are mainly distributed in hillside meadows, valley marshes, river meadows, alpine meadows, and hillside scrub meadows in Gansu, Qinghai, Sichuan, Yunnan, and other provinces at 2230–4700 m and 2000–5400 m, respectively. *S. superba* is a perennial herb that is mainly found in the sandy river valleys of the Gansu and Qinghai Provinces in China. Previous studies on *Saussurea* plants have focused on resource investigations [17], karyotyping [7,18], and chemical composition analyses [5], but chloroplast genomic studies on *S. katochaete*, *S. superba*, and *S. stella* have not been reported. Therefore, in this study, we assembled, annotated, and analyzed the chloroplast genomes of *S. katochaete*, *S. superba*, and *S. stella* using second- and third-generation high-throughput sequencing technologies and revealed their phylogenetic relationships. The results are expected to provide some references for the identification of populations, the identification of germplasm resources, and phylogenetic studies in the field of *Saussurea* plants.

## 2. Results

### 2.1. Characterization of the Saussurea Chloroplast Genome

**Figure 1:**
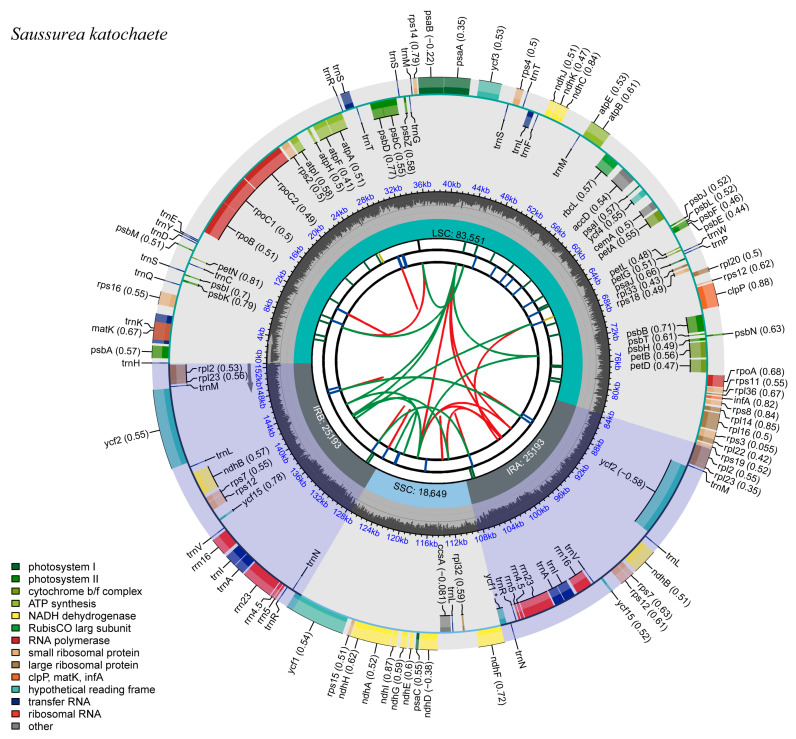
Chloroplast genome map of *S. katochaete*. The gray arrows indicate the direction of gene transcription. Genes in the inner circle are transcribed in a clockwise direction, while those in the outer circle are transcribed in a counter-clockwise direction.

**Figure 2:**
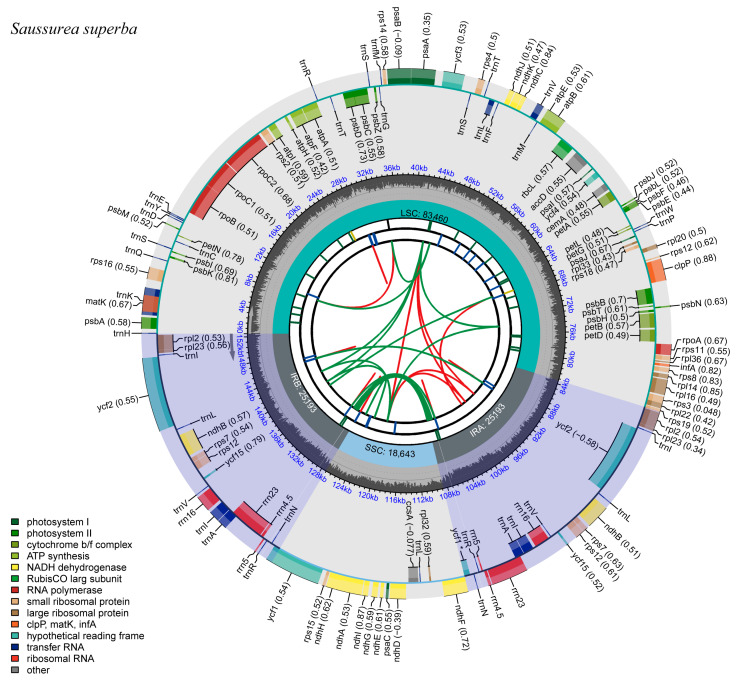
Chloroplast genome map of *S. superba*. The gray arrows indicate the direction of gene transcription. Genes in the inner circle are transcribed in a clockwise direction, while those in the outer circle are transcribed in a counter-clockwise direction.

**Figure 3:**
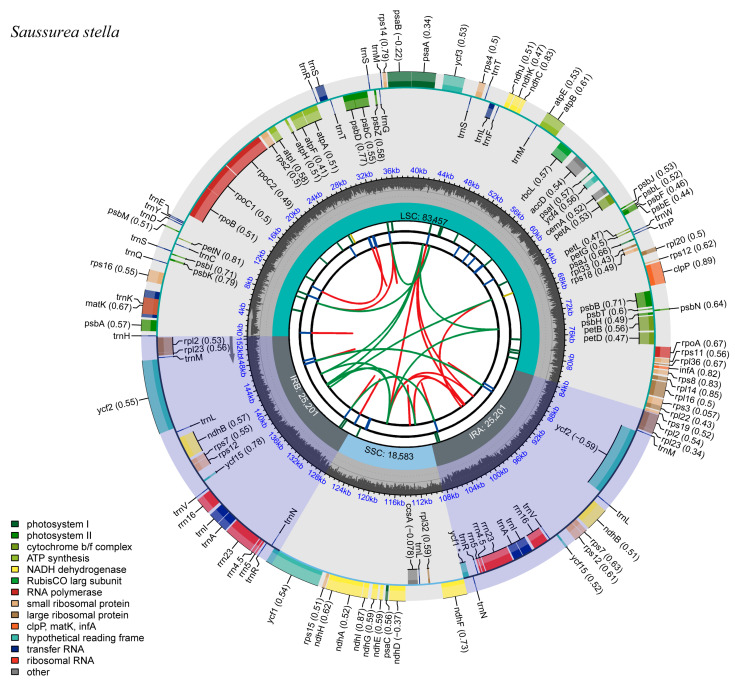
Chloroplast genome map of *S. stella*. The gray arrows indicate the direction of gene transcription. Genes in the inner circle are transcribed in a clockwise direction, while those in the outer circle are transcribed in a counter-clockwise direction.

The full-length sequences of the chloroplast genomes of the three *Saussurea* plants were 152,586 bp (*S. katochaete*), 152,490 bp (*S. superba*), and 152,442 bp (*S. stella*), respectively. Additionally, they were double-stranded loops with a typical quadripartite structure, as in most herbaceous plants. Two inverted repeat regions (IRs) separate the large single copy (LSC) and small single copy (SSC) regions (Figure 1, Figure 2 and Figure 3). In the IR regions of *S. katochaete*, *S. superba*, and *S. stella*, the IRa lengths were found to be 25,193 bp, 25,193 bp, and 25,201 bp, and the IRb lengths were 25,193 bp, 25,193 bp, and 25,201 bp, respectively. The lengths of the LSC regions were 83,551 bp, 83,460 bp, and 83,457 bp. The SSC region lengths were 18,649 bp, 18,644 bp, and 18,583 bp, respectively (Table 1). The GC contents of the three *Saussurea* plants were similar (37.68–37.69%), and the GC contents of the IR region (43.12–44.28%) were higher than those of the LSC region (35.80–35.94%) and the SSC region (31.39–31.40%) (Table 1). This might be related to the higher GC-rich rRNA and tRNA gene contents [14,19]. The AT content (62.31–62.32%) was greater than the GC content (37.68–37.69%), indicating the same characteristics as other plant chloroplast genomes, i.e., the AT bias was obvious [20].

### 2.2. Gene Annotation and Categorization Analysis

**Figure 4:**
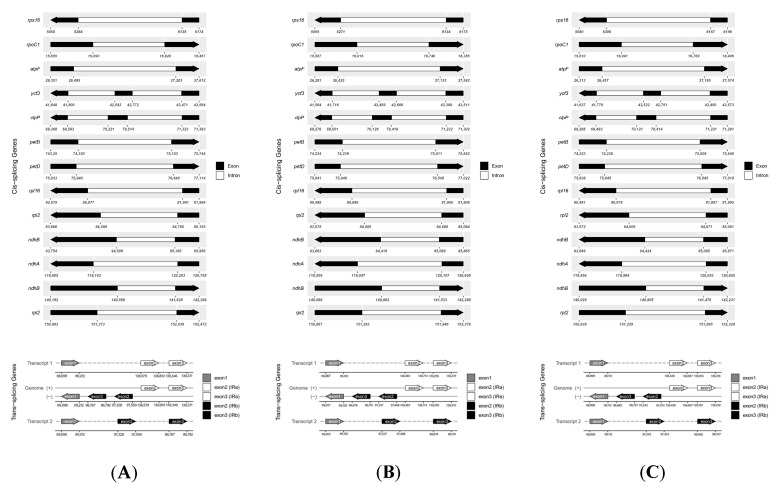
Schematic representation of cis-splicing and trans-splicing in *S. katochaete* (**A**), *S. superba* (**B**), and *S. stella* (**C**).

The results of the three *Saussurea* plants showed that the chloroplast genome of *S. katochaete* contains 128 genes, of which eighty-three are PCGs, eight are rRNA genes, and thirty-seven are tRNA genes (Table 2). The chloroplast genome of *S. superba* contains 124 genes, of which eighty are PCGs, eight are rRNA genes, and thirty-six are tRNA genes. The chloroplast genome of *S. stella* contains 127 genes, of which eighty-three are PCGs, eight are rRNA genes, and thirty-six are tRNA genes. The genes can be classified into four main categories based on their functions. The first category is the class of genes related to photosynthesis, including photosystem I, photosystem II, the cytochrome b/f complex, ATP synthase, and NADH dehydrogenase. The second category is the category of genes associated with one’s own inheritance, including the ribosomal protein class (SSU), ribosomal protein (LSU), RNA polymerase, RubisCO large subunit, transfer RNAs, and ribosomal RNAs. The third category contains genes associated with other syntheses, including protease, maturase, and translational initiation factors. The fourth category contains gene types with unknown functions. In addition, a total of 22 intron-containing genes were identified in the three *Saussurea* plants (Table 3, Table 4 and Table 5, Figure 4). Among these intron-containing genes, 18 genes (*atpF*, *clpP*, *ndhA*, *ndhB*, *petB*, *petD*, *rpl16*, *rpl2*, *rpl2*, *rpoC1*, *rps16*, *trnA-UGC*, *trnA-UGC*, *trnI-GAU*, *trnI-GAU*, *trnK-UUU*, *trnL-UAA,* and *trnS-CGA*) contain one intron, including thirteen PCGs and five tRNA genes, and two genes (*clpP* and *ycf3*) contain two introns. Two rps12 genes are trans-splicing genes.

### 2.3. Long Repeat Sequences and SSR Analyses

Long repetitive sequences comprise three types: forward (F), palindromic (P), and tandem (T) repeats. They may function to promote chloroplast genome rearrangements and can increase the population’s genetic diversity. A total of 90 unique long repetitive sequences were detected in the chloroplast genomes of three plants of the genus Saussurea (Table 6), of which 25 pairs of long repetitive sequences were found in S. katochaete, including one forward and twenty-four palindromic repetitions. Thirty-six and 29 pairs of long repetitive sequences were found in *S. superba*, and *S. stella* had 36 and 29 pairs of long repeat sequences, respectively, all of which were palindromic repeats (Table 6). The types of long repetitive sequences found in the chloroplast genomes of the three plant species of the genus Saussurea are almost all palindromic repeats with no tandem repeats, except for *S. katochaete*, which contains one forward repeat. Of these repeats, the shortest is only 30 bp in length, while the longest is as large as 4344 bp. The results indicate that the number, distribution, and length of the long repetitive sequences in the chloroplast genome of Saussurea plants are heterogeneous.

SSRs (Simple Sequence Repeats), i.e., Simple Repeat Sequences refer to a segment of DNA in the genome consisting of basic units of one to six nucleotides that are repeated many times. They are widely distributed among different locations of the genome. In addition, they are widely used as molecular markers in species identification and phylogenetic studies [19]. The annotation results showed that a total of 908 SSRs were identified, with the numbers of SSRs for *S. katochaete*, *S. superba*, and *S. stella* being 305, 303, and 300, respectively (Table 7). The average number of SSRs was about 303, with the highest number of SSRs being found in *S. katochaete* and the lowest number of SSRs being found in *S. stella*. Among them, 46, 45, and 43 SSR loci were identified in three *Saussurea* plants (Table 8, Table 9 and Table 10). Most of the SSR loci were located in the LSC region (37, 37, and 34 loci) of the *Saussurea* chloroplast genome, whereas relatively few SSR loci were located in the SSC region (4, 4, and 4 loci) and the IR region (5, 5, and 5 loci). In addition, the six types of SSRs identified were mononucleotide (36.12%), dinucleotide (50%), trinucleotide (5.07%), tetranucleotide (6.72%), pentanucleotide (1.32%), and hexanucleotide (0.77%). Dinucleotide and mononucleotide were the most predominant types of SSRs. The five major repetitive sequence types were A/T, AA/TT, AAA/TTT, AAAA/TTTT, and AAAAA/TTTTT.

### 2.4. Codon Usage Bias

The PCGs in the chloroplast genomes of *S. katochaete*, *S. superba*, and *S. stella* contained 21,408, 18,341, and 18,986 codons, respectively (Table 11). Of the amino acids encoded, the most encoded amino acid was leucine (Leu), which accounted for 10.49%, 10.33%, and 10.23% of all encoded amino acids, respectively, in the three plant types. The amino acid that was least encoded was cysteine (Cys), which accounted for only 1.07%, 1.03%, and 1.07% of all encoded amino acids, respectively, in the three plant types. This result is similar to that of a previous study on herbaceous plants [21]. Based on the RSCU values, the number of preferred codons in the chloroplast genomes of all three *Saussurea* plants was found to be 30 (RSCU > 1), and the number of non-preferred codons was found to be 32 (RSCU < 1). Among these, the 29 preferred codons, except for the UUG, which ended in G/C, ended in A/U. This indicates that the codons ending in the base of A/U are preferred codons, and codons ending in G/C are non-preferred codons [22], a result that may be related to the adaptive evolution of the plant chloroplast genomes [23]. This is in contrast to the non-preferred codons (RSCU < 1), which mostly end in G/C, suggesting that these types of codons occur less frequently in the *Saussurea* chloroplast genome. In addition, methionine (Met) and tryptophan (Trp) were encoded by only one codon, and both RSCUs were 1. The number of codons encoded by the rest of the codons ranged from two to six, and the results were in agreement with the findings of Chong et al. and Shi et al. [19,24]. The three termination codons detected were UAA, UAG, and UGA, with UAA being the most frequent, i.e., the termination codons were biased towards UAA.

### 2.5. IR Expansion and Contraction

**Figure 5:**
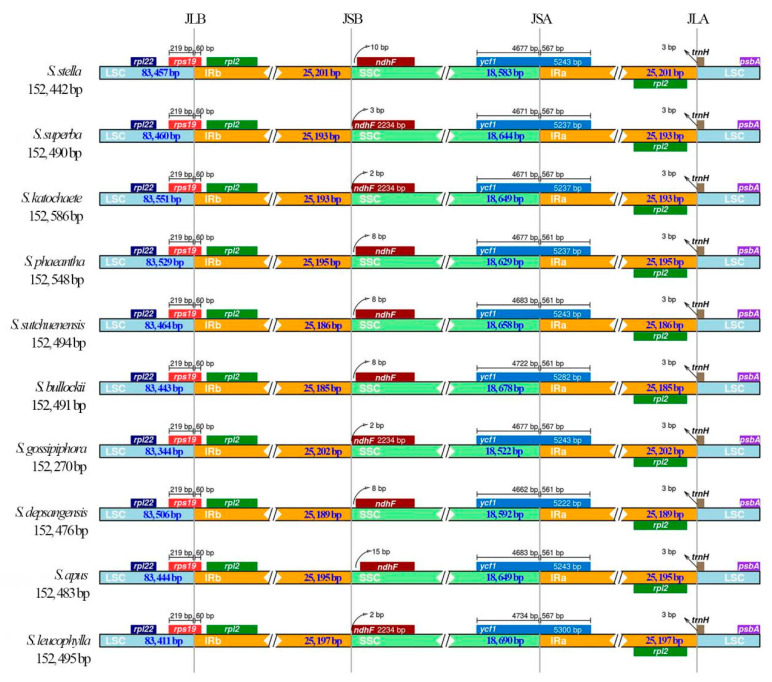
Comparison of the boundaries of the LSC, SSC, and IR regions of the chloroplast genomes of 10 species of *Saussurea*. Genes around the border are shown above and below the main line. JLB, JSB, JSA, and JLA represent the junctions of LSC/IRb, IRb/SSC, SSC/IRa, and IRa/LSC, respectively.

In this study, we comparatively analyzed the boundaries of the IR region of the chloroplast genomes of *S. katochaete*, *S. superba*, and *S. stella* with those of seven closely related species: *Saussurea phaeantha*, *Saussurea sutchuenensis*, *Saussurea apus*, *Saussurea depsangensis*, *Saussurea gossipiphora*, *Saussurea bullockii,* and *Saussurea leucophylla* (Figure 5). The results showed that the lengths of the chloroplast genomes of the 10 *Saussurea* plants range from 152,270 (*S. gossipiphora*) to 152,586 (*S. katochaete*) bp. The length of the IR region was found to be 25,185 (*S. bullockii*) to 25,202 (*S. gossipiphora*) bp. The length of the LSC region was found to be 83,344 (*S. gossipiphora*) to 83,551 (*S. katochaete*) bp, and the length of the SSC region was found to be 18,522 (*S. gossipiphora*) to 18,690 (*S. leucophylla*) bp. The chloroplast genes of the 10 species differed in length by 316 bp. The LSC region differed by 207 bp, the SSC region differed by 168 bp, and the IR region differed by 77 bp. The IR showed a high degree of conservatism with the LSC and SSC boundaries, and genes spanning or close to the boundaries of the IR and SC regions mainly included *rps19*, *rpl22*, *rpl2*, *ycf1*, *ndhF*, and *trnH*. Among them, *ndhF*, which is involved in photosynthesis, is located across the JSB boundary, 2–15 bp away from the JSB boundary. The *ycf1* gene crosses the JSA boundary and extends from 5222 (*S. depsangensis*) to 5300 (*S. leucopphylla*) bp towards the Ira region and from 18,522 (*S. gossipiphora*) to 18,690 (*S. leucophylla*) bp from the IRa region towards the SSC region. The *rps19* gene extends 6 bp towards the IRb region. The JLA boundary is located between *rpl2* and *trnH*.

### 2.6. Comparative Genome Analyses of Saussurea Species

**Figure 6:**
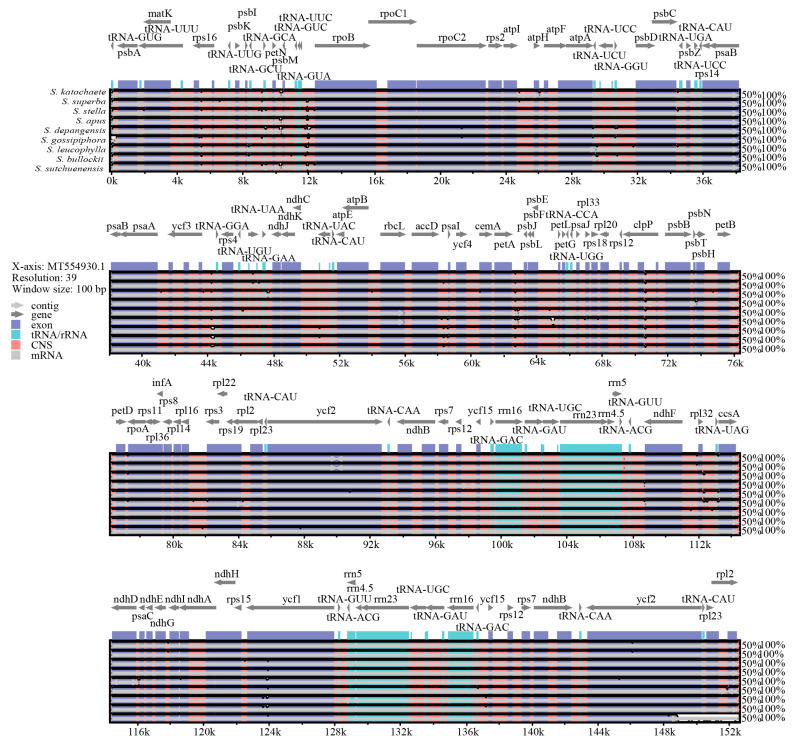
Comparative genomic analyses of 10 species of *Saussurea*. Gray arrows indicate the gene orientation, pink indicates non-coding sequences, purple indicates exons, light blue indicates rRNAs, and white bumps indicate different regions of chloroplast genes. The x-axis delineates the aligned nucleotide positions from the *S*. *phaeantha* chloroplast genome (MT554930.1), and the y-axis displays pairwise identity percentages, spanning from 50% to 100%.

**Figure 7:**
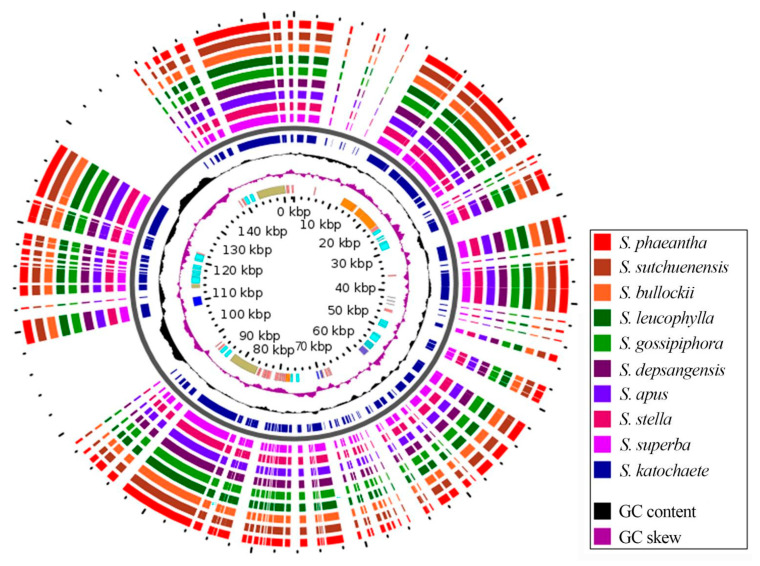
The BLAST Atlas results of chloroplast genomes of 10 *Saussurea* species. The CDS regions in the reference genome were BLASTed against the CDS regions in the query genomes, and the top hits were rendered in a genome map using GView. The purple and black wave charts represent the GC content and skew. The innermost slot on the map (orange) shows the CDS regions on the reference genome. The reference was *S. katochaete*. From the outside to the inside, they represent *S. phaeantha*, *S. sutchuenensis*, *S. bullockii*, *S. leucophylla*, *S. gossipiphora*, *S. depsangensis*, *S. apus*, *S. stella* and *S. superba*.

To understand the degree of sequence similarity between the chloroplast genomes of *S. katochaete*, *S. superba,* and *S. stella* and the other seven close relatives to *Saussurea*, the *S. phaeantha* chloroplast genome was used as the reference sequence for a full sequence comparison and analysis. The results show that the chloroplast genome sequences of the 10 species exhibit high levels of similarity or highly conserved genes, and no obvious gene rearrangements were found (Supplementary Figure S1, Figure 6). In comparison, the sequences of the LSC and SSC regions were found to be more variable than those of the IR regions (Figure 7), and the sequences of non-coding regions were more variable than those of the coding regions. Among these, several loci, such as *TRN-GCA-petN*, *rpl32-trnL-UAG*, and *ycf1*, showed lower levels of similarity.

### 2.7. Analysis of the Nucleotide Diversity

**Figure 8:**
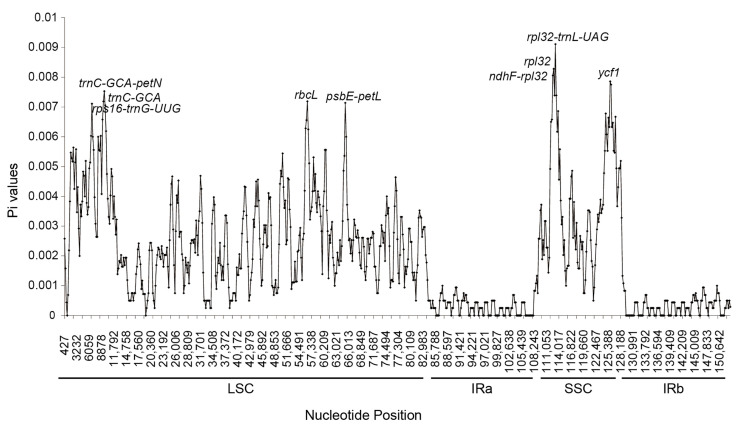
Analysis of the nucleotide diversity in the chloroplast genomes of 10 species of *Saussurea*. The X-axis and Y-axis show the positions of the midpoint of a window and the pi values, respectively.

A total of 1112 variable (polymorphic) sites were identified in 151,485 nucleotide sites, including 694 single variable sites (SVS) and 418 parsimony information sites (PIS). Two different types were observed under SVS: 690 for two variable sites (SV2V) and four for three variable sites (SV3V). Similarly, there were two types of PIS: four loci with two variants (PIS2V) and seven loci with three variants (PIS3V). In addition, to quantify the level of nucleotide polymorphisms, the chloroplast genomes of 10 *Saussurea* plant species were compared and analyzed using DNAsp software(Version: v.5.10.01). The results showed that the Pi values of nucleotide polymorphisms in the chloroplast genomes of the 10 *Saussurea* species varied from 0 to 0.00911, with a mean value of 0.00200. At least nine highly variable regions are included in the *Saussurea* chloroplast genomes, i.e., *rpl32-trnL-UAG*, *rpl32*, *ndhF-rpl32*, *ycf1*, *trnC-GCA-petN*, *trnC-GCA*, *rpcL*, *psbE-petL*, and *rpl16-trnG-UUG*. These genes or gene spacer regions have high variability (Pi > 0.007). The highest values of nucleotide variation (Pi) found were 0.00911, 0.00828, 0.00806, 0.00786, 0.00753, 0.00719, 0.00719, 0.00714, and 0.00711 (Figure 8). These can be used as a potential DNA barcode for *Saussurea* species.

### 2.8. Phylogenetic Analysis

**Figure 9:**
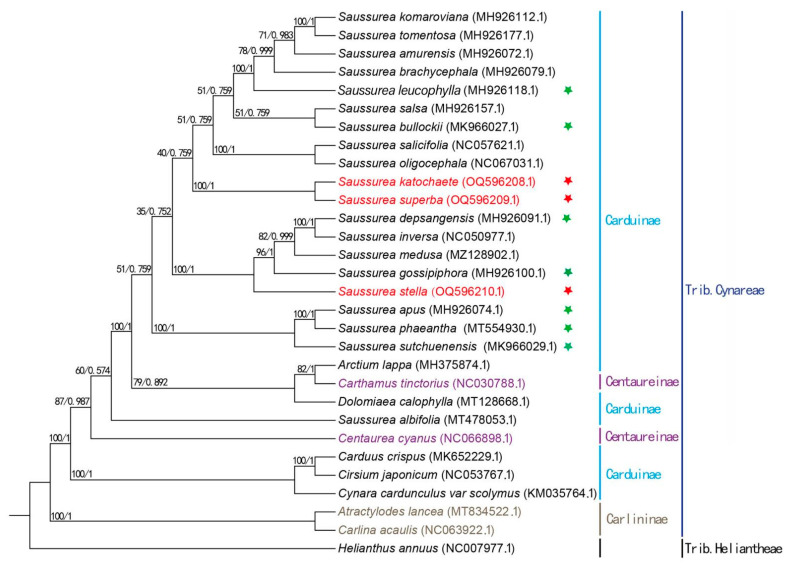
Phylogenetic analysis of the *Saussurea*. The red pentagrams represent the three species included in this study, and the green pentagrams represent the seven closely related species. The ML tree was reconstructed by IQ-TREE v.1.6.8. The numbers next to the branches represent ML bootstrap support values. The BI tree was reconstructed by MrBayes v. 3.2.6. The numbers next to the branches represent BI probability support values.

The phylogenetic trees constructed based on both the ML and BI methods are based on agreement (Figure 9). Twenty-nine species of the Trib. Cynareae clustered in the same large branch, except for the exotic taxa. The two Carlininae plants, *Atractylodes lancea* and *Carlina acaulis*, located at the base of the evolutionary tree, clustered into one branch. Secondly, *Carduus crispus*, *Cirsium japonicum*, and *Cynara cardunculus* of the Carduinae clustered into one branch. *Centaurea cyrdunculus* of Centaureinae clustered into one branch, while *Carthamus tinctorius* of Centaureinae and *Arctium lappa* of Carduinae, as well as *Dolomiaea calophylla* of Carduinae, grouped together in a single unit, distinguishing *S. albifolia* of Carduinae from 19 other plants of the same genus. In the present study, *S. stella* clustered with *S. depsagensis*, *S. inversa*, *S. medusa*, and *S. gossipihora*, while *S. katochaete* and *S. superba* clustered together in a single unit that was closest in kinship to *S. gossipiphora*. It can be seen that *S. katochaete* and *S. superba* are closely related, and both are relatively distantly related to *S. stella*.

## 3. Discussion

### 3.1. Chloroplast Genomic Characteristics and Sequence Variation in the Three Saussurea Species

In this study, the complete chloroplast genomes of three species of *Saussurea*—*S. katochaete*, *S. superba*, and *S. stella*. They were found to be similar to those of other herbaceous plants with typical double-stranded circular tetramer structures [16,25] and sizes of 152,561 bp, 151,452 bp, and 152,293 bp, respectively. Changes in the size of plant chloroplast genomes are mainly affected by the expansion and contraction of IR regions, variation in spacer regions, and gene loss. At the same time, these expansion and contraction events cause sequence disruptions of genes located at the edges of IR regions, ultimately resulting in the formation of pseudogenes. These pseudogenes do not participate in the protein-coding process, and their products are non-essential in the organism; hence, they are also known as non-functional genes [24,26,27]. However, some other studies have found that the nucleotide sequences of the pseudogenes are well-preserved, and therefore, they may not be non-functional genes, as traditionally perceived [28]. In this study, 124–128 genes were identified in the chloroplast genomes of three *Saussurea* plants. Their protein-coding genes, tRNA genes, rRNA genes, and intron genes showed extremely similar characteristics in terms of number, which explains, to some extent, why they have similar epimorphologies. Among them, two shared pseudogenes, rps19 and ycf1, were also identified in the gene sequence. These are commonly found in the chloroplast genomes of many angiosperms and herbaceous plants, such as *Lycium* (Solanaceae) [29], *Allium chrysanthum* (Amaryllidaceae J.St.-Hil.) [30], and *C*. *cardunculus* (Asteraceae) [31], among others. In recent years, it has been found that the *ycf1* gene may be involved in the process of photosynthesis and in responses to environmental changes in plants [32]. It is a key DNA barcode in plants [23], but expansion and contraction of the IR region may also lead to its fragmentation [26]. In addition, the contraction and expansion of the IR region result in the loss of the *ycf2* gene in *S. superba* and *S. stella*, suggesting that genetic differentiation may occur in the region of loss in *Saussurea* species and may also inhibit chloroplast genome enlargement in the *Saussurea* species to a certain extent [24,33].

A total of 22 intron-containing genes were identified in three plants of the genus *Saussurea*. These included 18 genes with one intron, two genes with two introns, and two trans-shear genes. These intron-containing genes can play important roles in regulating gene expression and improving agronomic traits in plants [34].

The determination of long repeated sequences and Simple Sequence Repeats (SSRs) is closely related to the genetic diversity of plants and the identification of molecular markers for germplasm resources [35]. Long repetitive sequences are usually found in the intergenic spacer (IGS) and intronic regions of plant chloroplast genomes [36]. In this study, the repetitive sequences observed in the chloroplast genomes of the three *Saussurea* plant species showed a very heterogeneous phenomenon in terms of the number, type, and length. With palindromic (P) repeats occurring at a very high frequency relative to forward (F) repeats, tandem (T) repeats were not detected in any of the three *Saussurea* plants, suggesting that there may be a certain degree of difference in the mutation frequency among the three species [35]. These long repetitive sequences play important roles in gene recombination and sequence structure variation [23] and can serve as potential indicators of differential identification between *Saussurea* species. SSRs are a class of short repetitive sequences, or Simple Sequence Repeats, found in the chloroplast genome. They are also known as microsatellites. They are prevalent in eukaryotes and prokaryotes and are closely related to gene expression and regulation [37]. These DNA sequences are widely involved in a wide range of life processes in plant cells, and because of their high level of polymorphism, they are often used in the fields of genetic diversity, species identification, and the development of molecular markers [21,23]. The three *Saussurea* plants included in this study, *S. katochaete*, *S. superba*, and *S. stella*, were identified as having 46, 45, and 43 SSR loci, respectively (Table 9, Table 10 and Table 11). Six types of SSRs were included, i.e., mononucleotide, dinucleotide, trinucleotide, tetranucleotide, pentanucleotide, and hexanucleotide, which are mostly located in the non-coding region. Mononucleotide (A/T) is the predominant type of repeat, a similar result to studies on the predominance of A/T in the base composition of SSRs in other herbaceous plants, such as *Thalictrum cirrhosum* (Ranunculaceae) [38] and *Themeda japonica* (Gramineae) [39]. This A/T-dominant repeat type is thought to be a widespread phenomenon in the chloroplast genomes of higher plants, and the fact that preferred codons predominantly end in A/U causes this bias. Previous studies have suggested that this may be related to natural selection and genetic mutation [40]. In addition, we identified nine highly variable genes or intergenic regions (*rpl32-trnL-UAG*, *rpl32*, *ndhF-rpl32*, *ycf1*, *trnC-GCA-petN*, *trnC-GCA*, *rpcL*, *psbE-petL*, and *rpl16-trnG-UUG*), and it is clear that the sequence variability is significantly higher in the LSC region and the SSC region than in the IRs. This may be caused by certain duplicated genes within the IR region preventing mutations from occurring [40]. Some of these rare SSR loci as well as highly variable regions can be used as potential molecular markers in the fields of intraspecific genetic variation, population identification, species evolution, and phylogenetic studies in plants, e.g., *rps8*, *rpl16*, *PsbE-petL*, and *ndhF-rpl32* have been demonstrated to be useful in *Phoenix dancong* (Camellia sinensis) [41], *Oryza sativa* (Gramineae) [42], and some Lauraceae plants (*Machilus yunnanensis*, *Machilus balansae*) [43] in terms of population identification and phylogenetic analyses, demonstrating high levels of resolution.

Codon preferences in plant chloroplast genomes can reveal phylogenetic relationships across species or within the same species, and mutation and natural selection are closely linked to codon preferences in genes [44]. For example, the codon preferences of some Euphorbiaceae [45] plants are mainly influenced by natural selection, and those of *Oncidium Gower Ramsey* [46], an ornamental flower, are mainly influenced by mutations. It has also been suggested that the factors affecting codon preferences in the chloroplast genome are complex and diverse and are not solely influenced by a single natural selection or mutation [47]. In this study, codons ending in A/U were dominant in the chloroplast genomes of the three *Saussurea* plants, and the RSCU values were close to each other, with the number of preferred codons being 30 (RSCU > 1) and the number of non-preferred codons being 32 (RSCU < 1). This suggests that the chloroplast genomes of *Saussurea* plants are relatively conserved, and they have formed a unique codon use system that distinguishes them from other species during the evolutionary and developmental processes. In addition, the three *Saussurea* species showed a higher preference for the codon UUA, which encodes an amino acid type of Leu, than for other codons with RSCUs as high as 1.95, 2.1, and 2.05, respectively. This suggests that the *trnL-UAA* gene may have exerted an important influence on the evolutionary development of *Saussurea* species.

### 3.2. Phylogenetic Analyses in Saussurea Species

Although the chloroplast genome dataset is still considered to have many shortcomings, the gene sequences are still important for revealing phylogenetic relationships between or within species because they include many important information sites [24,33,48]. The phylogenetic tree of Saussurea plants, like those of other reported species [24], has similar topological structures, suggesting that many closely related species within the genus may have originated from a common ancestor. The flanking genes of the JLB, JSB, JSA, and JLA borders of the three species in this study, *S. katochaete*, *S. superba*, and *S. stella*, were the same, being the *rpl22*, *rps19, rpl2*, *ndhF*, *ycf1,* and *psbA* genes. With the exception of minor differences in the amplification lengths of the genes at the JSB boundary, the amplification lengths were consistent across the remaining boundaries, suggesting close phylogenetic relationships among these three *Saussurea* species. After they diverged from a common ancestor, *S. stella*, *S. depsagensis*, *S. inversa*, *S. medusa*, and *S. gossipihora* clustered together in a separate monophyletic group. Additionally, *S. katochaete* and *S. superba* evolved into a sisterhood. Obviously, *S. katochaete* and *S. superba* are more closely related compared to *S. stella*.

*Saussurea* was first established in 1810, and it is distributed in Asia and Europe. In China, it is mainly distributed in the high-altitude areas of southwest and northwest China. Due to the wide distribution of the genus, the great variety of species, and the unclear morphological differentiation among species, coupled with the fact that the classification of the genus is mainly based on phenotypic characteristics, such as the subtending leaf of capitulate, etc., its taxonomic study and interspecific identification have been controversial [4,49]. Since the introduction of Mendel’s laws of inheritance in 1865, researchers have generally believed that the evolutionary process of plants can be fully explained by applying Mendel’s laws of inheritance. However, studies on some higher plants proved in 1909 that certain variable traits in plant evolution may be closely related to chloroplasts rather than being caused by Mendelian modes of inheritance [50]. It was not until the 1960s that Sager and Ishida’s studies further confirmed that there is indeed a unique class of genetic material in the plant chloroplast genome that is capable of influencing certain traits in plants [9]. Phylogenetic analyses of *Saussurea* plants in the present study revealed a high frequency of *Saussurea* species differentiation among different species and great morphological differences among many different species, which may also be related to certain genetic materials in the chloroplasts.

## 4. Materials and Methods

### 4.1. Plant Samples, DNA Extraction, and Sequencing

Leaves from *S. katochaete*, *S. superba*, and *S. stella* used for this study were collected from Yushu City, Yushu Tibetan Autonomous Prefecture, Qinghai Province, China (N: 96°29′35″, E: 33°18′58″, H: 4210 m), Menyuan County, Haibei Tibetan Autonomous Prefecture, Qinghai Province, China (N: 37°22′35″, E: 101°37′21″, H: 3276 m), and Zaduo County, Yushu Tibetan Autonomous Prefecture, Qinghai Province, China (N: 95°49′23″, E: 32°47′38″, H: 4601 m). DNA samples were extracted by the classical CTAB method, and the DNA quality was detected by 1% agarose gel electrophoresis. DNA was precisely quantified by Qubit. The constructed libraries were sequenced using Illumina NovaSeq, an Illumina high-throughput sequencing platform.

### 4.2. Genome Assembly and Annotation

Genome splicing was performed using Flye (version: v.2.9; parameters: meta-plasmids) software, and the splicing results were compared with a close reference genome using Blastn (version: 2.12.0+; parameters: evaluate 1 × 10^−5^) based on a comparison to determine the candidate sequence assembly results. The genomic linkages of chloroplasts were determined based on the depth of sequence sequencing, the read comparison situation, and the comparison situation with closely related species. The connected sequences, if containing gaps (containing N sequences), were further hole-patched using Gapcloser (Version: 1.12) to obtain the final splicing results.

The functional annotation of the chloroplast genome included coding gene predictions and non-coding RNA annotations (rRNA and tRNA annotations). Gene annotations were performed using the chloroplast-specific annotation software CPGAVAS2 (http://47.96.249.172:16019/analyzer/annotate accessed on 2 August 2023).

### 4.3. Genome Structure Analyses

SSRs (Simple Sequence Repeats) are segments of DNA in the genome consisting of basic units of one to six nucleotides repeated many times that are widely distributed in different locations in the genome, and the flanking sequences are usually single sequences that are strongly conserved. SSRs were performed on chloroplasts using MISA (version: v2.1; default parameters; corresponding to the minimum number of replicates for each replicate unit (unit size): 1-8 2-8 2-8 2-8 2-4 3-4 4-4 4-3 5-3 6-3, respectively), as described in (http://pgrc.ipk-gatersleben.de/misa/misa.html accessed on 16 September 2023).

We used vmatch (http://www.vmatch.de/ accessed on 7 August 2023; parameter: minimal repeat size 30 bp) to find scattered long repeat sequence fragments in the chloroplast genome. Codon usage bias, or relative synonymous codon usage (RSCU), is an assessment of the preference for the use of synonymous codons. The value is equal to the ratio of the actual observed value of synonymous codons to the average expected value of synonymous codon usage. If there is no preference for codon usage, the RSCU value is 1; if the codon is used more frequently than other synonymous codons, its RSCU value is greater than 1; if the opposite is true, the RSCU value is less than 1. The frequency of relative synonymous codon usage was statistically estimated for codons greater than 300 in length and with “ATG”, “TTG”, “CTG”, “ATT”, “ATC”, “GTG”, and “ATA” as start codons. With “ATT”, “ATC”, “GTG”, and “ATA” as start codons, the use of “TGA”, “TAG”, and “TAA” as stop codons was analyzed for codon usage bias using CodonW (Version: 1.4.4). Sequences were analyzed for codon preference using CodonW (Version: 1.4.4).

### 4.4. Genome Comparison and Nucleotide Variation Analysis

The LSC, SSC, and IR region boundaries of the *Saussurea* chloroplast gene regions and adjacent genes were visualized and analyzed using the IRscope online analysis tool (Amiryousefi et al., 2018). The whole sequence of the *Saussurea* chloroplast genome was compared and visualized using the mVISTA online tool (https://genome.lbl.gov/vista/index.shtml accessed on 8 August 2023) in shuffle-LAGAN mode using *S. phaeantha* as a reference sequence. The corresponding genome sequences were compared using MAFFT software (Version: v7.487). A chloroplast genome polymorphism analysis (nucleotide diversity, Pi) was performed using DNAsp software (Version: v.5.10.01) with a window length of 800 bp and a glide step of 200 bp.

### 4.5. Phylogenetic Analyses

To clarify the phylogenetic positions of *S. katochaete*, *S. superba*, and *S. stella* in *Saussurea*, 20 *Saussurea* plants and nine other Compositae, Trib. Cynareae plants were studied, and the Compositae, Trb. Heliantheae, Helianthus, and *Helianthus annuus* were used as an outgroup (Table 12). They were analyzed by a multiple sequence comparison using MAFFT software (Version: v7.487), and then IQ-TREE software (Version: v.1.6.8.) was used to construct an ML tree (maximum likelihood method) using the TVM+F+I+G4 model and a BI tree (Bayesian method) using MrBayes (Version: v. 3.2.6.) using the GTR+I+G model with 2,000,000 generations and a sampling frequency of 100. The visualization was performed using ITOL software (http://itol.embl.de/ accessed on 17 August 2023).

## 5. Conclusions

In this study, we assembled, annotated, and analyzed the whole chloroplast genomes of three species of *Saussurea* and explored their genomic features and phylogenetic relationships with other closely related species. The results show that the three *Saussurea* plants, similar to other herbaceous plants, have relatively conserved genome structures, abundant SSR loci, and some highly variable genes or gene intergenic regions. This provides an important basis for the population identification and phylogenetic study of *Saussurea* plants. The degree of sequence variation in the LSC and SSC regions of the genome was significantly higher than that in the IR region. The *trnL-UAA* gene may have had an important influence on the evolutionary development of species of *Saussurea*. In addition, phylogenetic trees constructed based on ML and BI indicated that *S. katochaete* and *S. superba* are more closely related compared to *S. stella*. Additionally, the great variation among different species of *Saussurea* might be related to certain genetic materials in the chloroplast genome. The results of this study are important in terms of population identification, phylogeny, genetic variation, species evolution, and the identification of germplasm resources in *Saussurea* plants.

## Figures and Tables

**Table 1:** Chloroplast genome base composition of three Saussurea species.

Species	Region	Lengths	A (%)	T (%)	C (%)	G (%)	AT (%)	GC (%)
*S. katochaete*	Total	152,561	31.05	31.27	18.55	19.13	62.32	37.68
	LSC	83,559	31.94	32.26	17.57	18.24	64.20	35.80
	IRA	25,181	28.26	28.62	20.71	22.41	56.87	43.13
	SSC	18,649	34.07	34.54	14.84	16.55	68.61	31.39
	IRB	25,172	28.63	28.25	22.41	20.71	56.88	43.12
*S. superba*	Total	151,856	31.04	31.27	18.56	19.13	62.31	37.69
	LSC	87,755	31.79	32.27	17.67	18.27	64.06	35.94
	IRA	20,296	28.04	27.69	21.02	23.26	55.72	44.28
	SSC	18,643	34.08	34.52	14.84	16.56	68.6	31.40
	IRB	20,287	27.69	28.03	23.25	21.03	55.72	44.28
*S. stella*	Total	152,368	31.05	31.27	18.56	19.12	62.32	37.68
	LSC	83,430	31.94	32.26	17.57	18.22	64.2	35.80
	IRA	25,174	28.25	28.62	20.7	22.42	56.88	43.12
	SSC	18,581	34.09	34.52	14.86	16.53	68.61	31.39
	IRB	25,183	28.63	28.24	22.43	20.70	56.87	43.13

IRA and IRB represent two inverted repeat regions; LSC represents the large single copy region; and SSC represents the small single copy region.

**Table 2:** Chloroplast genome annotation and classification analysis of *S. katochaete*, *S. superba*, and *S. stella*.

Gene Function	Gene Category	Gene Name		
		*S. katochaete*	*S.* *superba*	*S. stella*
Photosynthesis	Photosystem I	*psaC, psaB, psaI, psaA, psaJ*	*psaA, psaJ, psaC, psaI, psaB*	*psaC, psaJ, psaI, psaA, psaB*
	Photosystem II	*psbE, psbD, psbT, psbZ, psbI, psbC, psbH, psbJ, psbA, psbN, psbM, psbB, psbK, psbF*	*psbM, psbZ, psbH, psbT, psbA, psbN, psbJ, psbK, psbC, psbD, psbI, psbE, psbB, psbF*	*psbM, psbH, psbN, psbB, psbZ, psbD, psbF, psbK, psbA, psbC, psbE, psbJ, psbI, psbT*
	Cytochrome b/f complex	*petB* *, *petD* *, *petL, petN, petA, petG*	*petD* *, *petB* *, *petL, petG, petN*	*petN, petB* *, *petD* *, *petA, petG, petL*
	ATP synthase	*atpA, atpE, atpH, atpF* *, *atpB, atpI*	*atpF* *, *atpB, atpE, atpI, atpH, atpA*	*atpH, atpE, atpB, atpI, atpF* *, *atpA*
	NADH dehydrogenase	*ndhC, ndhH, ndhB* *, *ndhK, ndhA* *, *ndhI, ndhD, ndhG, ndhE, ndhF, ndhJ*	*ndhA* *, *ndhJ, ndhG, ndhH, ndhE, ndhF, ndhC, ndhD, ndhI, ndhK*	*ndhH, ndhJ, ndhE, ndhG, ndhB* *, *ndhK, ndhF, ndhD, ndhA* *, *ndhC, ndhI*
Self-inheritance	Ribosomal proteins (SSU)	*rps3, rps11, rps16* *, *rps12, rps4, rps14, rps18, rps8, rps7, rps19, rps15, rps2*	*rps18, rps11, rps3, rps2, rps15, rps7, rps8, rps12, rps19, rps4, rps14, rps16* *	*rps3, rps2, rps19, rps4, rps18, rps14, rps11, rps15, rps12, rps16* *, *rps7, rps8*
	Ribosomal proteins (LSU)	*rpl14, rpl16* *, *rpl2* *, *rpl36, rpl22, rpl33, rpl23, rpl20, rpl32*	*rpl36, rpl32, rpl33, rpl20, rpl22, rpl23, rpl2* *, *rpl14, rpl16* *	*rpl2* *, *rpl23, rpl14, rpl22, rpl36, rpl16* *, *rpl33, rpl20, rpl32*
	RNA polymerase	*rpoB, rpoC2, rpoC1* *, *rpoA*	*rpoB, rpoC1* *, *rpoA, rpoC2*	*rpoA, rpoC2, rpoC1* *, *rpoB*
	RubisCO large subunit	*rbcL*	*rbcL*	*rbcL*
	Transfer RNAs	*trnL-UAG, trnQ-UUG, trnS-GCU, trnA-UGC* *, *trnN-GUU, trnH-GUG, trnS-GGA, trnR-ACG, trnV-UAC, trnV-GAC, trnR-UCU, trnD-GUC, trnM-CAU, trnE-UUC, trnC-GCA, trnI-CAU, trnL-CAA, trnK-UUU* *, *trnG-GCC, trnG-UCC, trnL-UAA* *, *trnI-GAU* *, *trnF-GAA, trnfM-CAU, trnT-GGU, trnP-UGG, trnT-UGU, trnW-CCA, trnY-GUA*	*trnI-CAU, trnA-UGC* *, *trnT-GGU, trnG-GCC, trnL-CAA, trnL-UAG, trnI-GAU* *, *trnQ-UUG, trnV-UAC, trnfM-CAU, trnD-GUC, trnK-UUU* *, *trnN-GUU, trnP-UGG, trnR-UCU, trnV-GAC, trnM-CAU, trnR-ACG, trnC-GCA, trnY-GUA, trnS-GCU, trnW-CCA, trnL-UAA* *, *trnE-UUC, trnG-UCC, trnT-UGU, trnF-GAA, trnS-GGA*	*trnW-CCA, trnR-UCU, trnfM-CAU, trnG-GCC, trnL-CAA, trnK-UUU* *, *trnE-UUC, trnD-GUC, trnM-CAU, trnP-UGG, trnV-GAC, trnT-GGU, trnN-GUU, trnF-GAA, trnS-GCU, trnY-GUA, trnQ-UUG, trnS-GGA, trnG-UCC, trnR-ACG, trnI-GAU* *, *trnI-CAU, trnT-UGU, trnC-GCA, trnA-UGC* *, *trnL-UAG, trnV-UAC, trnL-UAA* *
	Ribosomal RNAs	*rrn16S, rrn23S, rrn4.5S, rrn5S*	*rrn4.5S, rrn23S, rrn5S, rrn16S*	*rrn16S, rrn23S, rrn5S, rrn4.5S*
Other genes	Protease	*clpP* **	*clpP* **	*clpP* **
	Maturase	*matK*	*matK*	*matK*
	Translational initiation factor	*infA*	*infA*	*infA*
	Other genes	*accD, cemA, ccsA*	*accD, ccsA, cemA*	*ccsA, cemA, accD*
Genes of unknown function	Hypothetical chloroplast reading frames (ycf)	*ycf4, ycf3* **, *ycf2, ycf15, ycf1*	*ycf3* **, *ycf1, ycf4, ycf15*	*ycf4, ycf15, ycf1, ycf3* **

* Gene with one intron; ** gene with two introns.

**Table 3:** The lengths of the introns and exons for the splitting genes of the chloroplast genome of *S. katochaete*.

Gene	Strand	Start	End	Exon I	Intron I	Exon II	Intron II	Exon III
trnK-UUU	−	1738	4271	38	2460	36		
rps16	−	5058	6174	40	850	227		
rpoC1	+	15,659	18,457	432	729	1638		
atpF	+	26,351	27,612	145	707	410		
trnS-CGA	−	29,679	30,461	31	692	60		
ycf3	−	41,648	43,594	124	698	230	742	153
trnL-UAA	+	46,477	47,021	35	460	50		
clpP	−	69,368	71,393	71	808	294	627	226
petB	+	74,325	75,744	6	772	642		
petD	+	75,933	77,114	8	699	475		
rpl16	−	80,579	81,999	9	1013	399		
rpl2	−	83,666	85,155	391	665	434		
ndhB	−	93,754	95,956	777	670	756		
trnI-GAU	+	101,488	102,500	33	940	40		
trnA-UGC	+	102,565	103,458	37	821	36		
ndhA	−	118,605	120,755	553	1059	539		
trnA-UGC	−	132,680	133,573	37	821	36		
trnI-GAU	−	133,638	134,650	33	940	40		
ndhB	+	140,182	142,384	777	670	756		
rpl2	+	150,983	152,472	391	665	434		

**Table 4:** The lengths of the introns and exons for the splitting genes of the chloroplast genome of *S. superba*.

Gene	Strand	Start	End	Exon I	Intron I	Exon II	Intron II	Exon III
trnK-UUU	−	1738	4272	37	2462	36		
rps16	−	5059	6175	42	862	213		
rpoC1	+	15,587	18,385	430	729	1640		
atpF	+	26,281	27,542	145	707	410		
ycf3	−	41,564	43,511	124	699	230	742	153
trnL-UAA	+	46,393	46,937	37	458	50		
trnV-UAC	−	50,688	51,336	38	574	37		
clpP	−	69,276	71,302	71	812	291	627	226
petB	+	74,234	75,652	6	771	642		
petD	+	75,841	77,022	8	699	475		
rpl16	−	80,488	81,908	9	1013	399		
rpl2	−	83,575	85,064	397	662	431		
ndhB	−	93,663	95,865	777	670	756		
trnI-GAU	+	101,397	102,409	43	935	35		
trnA-UGC	+	102,474	103,367	38	821	35		
ndhA	−	118,509	120,659	553	1059	539		
trnA-UGC	−	132,584	133,477	38	821	35		
trnI-GAU	−	133,542	134,554	43	935	35		
ndhB	+	140,086	142,288	777	670	756		
rpl2	+	150,887	152,376	397	662	431		

**Table 5:** The lengths of the introns and exons for the splitting genes of *S. stella*.

Gene	Strand	Start	End	Exon I	Intron I	Exon II	Intron II	Exon III
trnK-UUU	−	1764	4294	38	2457	36		
rps16	−	5080	6196	40	850	227		
rpoC1	+	15,610	18,406	432	727	1638		
atpF	+	26,313	27,574	145	707	410		
trnS-CGA	−	29,640	30,422	31	692	60		
ycf3	−	41,627	43,573	124	698	230	742	153
trnL-UAA	+	46,487	47,013	35	442	50		
clpP	−	69,268	71,291	71	806	294	627	226
petB	+	74,223	75,649	6	779	642		
petD	+	75,838	77,019	8	699	475		
rpl16	−	80,481	81,905	9	1017	399		
rpl2	−	83,572	85,061	391	665	434		
ndhB	−	93,669	95,871	777	670	756		
trnI-GAU	+	101,403	102,414	33	939	40		
trnA-UGC	+	102,479	103,372	37	821	36		
ndhA	−	118,456	120,605	553	1058	539		
trnA-UGC	−	132,528	133,421	37	821	36		
trnI-GAU	−	133,486	134,497	33	939	40		
ndhB	+	140,029	142,231	777	670	756		
rpl2	+	150,839	152,328	391	665	434		

**Table 6:** Statistics for the long repetitive sequences in the chloroplast genomes of *S. katochaete*, *S. superba*, and *S. stella*.

*S. katochaete*		*S. superba*		*S. stella*	
Type	Length	Type	Length	Type	Length
F	46	P	1222	P	162
P	433	P	1581	P	702
P	2813	P	425	P	2379
P	4344	P	656	P	1590
P	811	P	329	P	2898
P	2161	P	100	P	442
P	2917	P	558	P	658
P	527	P	776	P	1747
P	2340	P	844	P	2720
P	795	P	510	P	90
P	266	P	113	P	1692
P	436	P	2858	P	419
P	45	P	1189	P	1085
P	863	P	691	P	829
P	1001	P	316	P	176
P	34	P	527	P	311
P	31	P	1475	P	863
P	39	P	612	P	47
P	1982	P	36	P	776
P	1675	P	52	P	1004
P	1710	P	39	P	323
P	42	P	735	P	935
P	48	P	1141	P	1676
P	34	P	358	P	474
P	30	P	500	P	1250
		P	482	P	42
		P	3475	P	48
		P	39	P	34
		P	501	P	30
		P	1194		
		P	813		
		P	438		
		P	42		
		P	48		
		P	34		
		P	30		

“F” for forward repeat, and “P” for palindromic repeat.

**Table 7:** SSR analysis of the chloroplast genomes of *S. katochaete*, *S. superba*, and *S. stella*.

Kind	Repetitive Sequence	*S. katochaete*		*S. superba*		*S. stella*	
		Quantity	Total	Quantity	Total	Quantity	Total
Mononucleotide	A/T	109	111	106	107	107	110
	C/G	2		1		3	
Dinucleotide	AA/TT	109	153	106	149	107	152
	AC/GT	2		2		2	
	AG/CT	19		19		19	
	AT/AT	20		20		20	
	CC/GG	2		1		3	
	CG/CG	1		1		1	
Trinucleotide	AAA/TTT	10	15	13	17	8	14
	AAG/CTT	1		1		1	
	AAT/ATT	3		3		4	
	CCC/GGG	1		/		1	
Tetranucleotide	AAAA/TTTT	10	21	13	22	8	18
	AAAC/GTTT	1		1		1	
	AAAG/CTTT	2		2		2	
	AAAT/ATTT	3		2		3	
	AATC/ATTG	2		2		1	
	ATAT/ATAT	2		2		2	
	CCCC/GGGG	1		/		1	
Pentanucleotide	AAAAA/TTTTT	3	4	4	5	2	3
	ACTAT/AGTAT	1		1		1	
Hexanucleotide	AAAAAA/TTTTTT	/	1	1	3	2	3
	AATAGG/ATTCCT	1		1		1	
	AGCAGG/CCTGCT	/		1		/	
			305		303		300

“/” means no such repeat type.

**Table 8:** SSRs in the chloroplast genome of *S. katochaete*.

ID	SSR nr.	SSR Type	SSR	Size	Start	End
OQ596208.1_Saussurea_katochaete	1	p4	(ATAA)3	12	1956	1967
OQ596208.1_Saussurea_katochaete	2	p1	(A)10	10	4340	4349
OQ596208.1_Saussurea_katochaete	3	p1	(C)14	14	5312	5325
OQ596208.1_Saussurea_katochaete	4	p1	(A)10	10	8239	8248
OQ596208.1_Saussurea_katochaete	5	p1	(A)10	10	9425	9434
OQ596208.1_Saussurea_katochaete	6	p1	(A)10	10	13,062	13,071
OQ596208.1_Saussurea_katochaete	7	p1	(A)10	10	18,106	18,115
OQ596208.1_Saussurea_katochaete	8	p2	(TA)5	10	18,329	18,338
OQ596208.1_Saussurea_katochaete	9	p2	(AT)5	10	19,325	19,334
OQ596208.1_Saussurea_katochaete	10	p6	(TATTCC)3	18	21,289	21,306
OQ596208.1_Saussurea_katochaete	11	p1	(T)10	10	22,923	22,932
OQ596208.1_Saussurea_katochaete	12	p1	(T)10	10	25,711	25,720
OQ596208.1_Saussurea_katochaete	13	p2	(AT)6	12	26,269	26,280
OQ596208.1_Saussurea_katochaete	14	p3	(TAA)4	12	30,743	30,754
OQ596208.1_Saussurea_katochaete	15	p5	(ACTAT)4	20	30,766	30,785
OQ596208.1_Saussurea_katochaete	16	p3	(TTC)4	12	34,221	34,232
OQ596208.1_Saussurea_katochaete	17	p1	(A)15	15	34,408	34,422
OQ596208.1_Saussurea_katochaete	18	p1	(T)11	11	34,428	34,438
OQ596208.1_Saussurea_katochaete	19	p1	(A)14	14	43,704	43,717
OQ596208.1_Saussurea_katochaete	20	p1	(T)11	11	46,147	46,157
OQ596208.1_Saussurea_katochaete	21	p1	(T)12	12	49,809	49,820
OQ596208.1_Saussurea_katochaete	22	p1	(T)13	13	54,162	54,174
OQ596208.1_Saussurea_katochaete	23	p3	(AAT)4	12	58,457	58,468
OQ596208.1_Saussurea_katochaete	24	p1	(T)10	10	58,809	58,818
OQ596208.1_Saussurea_katochaete	25	p1	(T)11	11	62,716	62,726
OQ596208.1_Saussurea_katochaete	26	p1	(T)17	17	64,541	64,557
OQ596208.1_Saussurea_katochaete	27	p1	(A)10	10	64,971	64,980
OQ596208.1_Saussurea_katochaete	28	p2	(TA)7	14	67,296	67,309
OQ596208.1_Saussurea_katochaete	29	p4	(TATT)3	12	67,354	67,365
OQ596208.1_Saussurea_katochaete	30	p1	(A)10	10	68,572	68,581
OQ596208.1_Saussurea_katochaete	31	p3	(ATA)4	12	70,641	70,652
OQ596208.1_Saussurea_katochaete	32	p1	(T)10	10	70,932	70,941
OQ596208.1_Saussurea_katochaete	33	p1	(A)12	12	77,280	77,291
OQ596208.1_Saussurea_katochaete	34	p1	(T)10	10	77,528	77,537
OQ596208.1_Saussurea_katochaete	35	p1	(T)15	15	79,960	79,974
OQ596208.1_Saussurea_katochaete	36	p1	(T)10	10	80,549	80,558
OQ596208.1_Saussurea_katochaete	37	p4	(TTTC)3	12	81,742	81,753
OQ596208.1_Saussurea_katochaete	38	p1	(A)12	12	107,033	107,044
OQ596208.1_Saussurea_katochaete	39	p1	(T)13	13	107,495	107,507
OQ596208.1_Saussurea_katochaete	40	p4	(TTTA)3	12	111,711	111,722
OQ596208.1_Saussurea_katochaete	41	p4	(AGAA)3	12	119,547	119,558
OQ596208.1_Saussurea_katochaete	42	p4	(AATC)3	12	119,954	119,965
OQ596208.1_Saussurea_katochaete	43	p4	(GATT)3	12	124,374	124,385
OQ596208.1_Saussurea_katochaete	44	p4	(CAAA)3	12	127,060	127,071
OQ596208.1_Saussurea_katochaete	45	p1	(A)13	13	128,631	128,643
OQ596208.1_Saussurea_katochaete	46	p1	(T)12	12	129,094	129,105

**Table 9:** SSRs in the chloroplast genome of *S. superba*.

ID	SSR nr.	SSR Type	SSR	Size	Start	End
OQ596209.1_Saussurea_superba	1	c	(ATAA)3(T)10	22	1956	1977
OQ596209.1_Saussurea_superba	2	p1	(T)10	10	3789	3798
OQ596209.1_Saussurea_superba	3	p1	(C)14	14	5313	5326
OQ596209.1_Saussurea_superba	4	p1	(A)10	10	8230	8239
OQ596209.1_Saussurea_superba	5	p1	(A)10	10	12,990	12,999
OQ596209.1_Saussurea_superba	6	p1	(A)10	10	18,034	18,043
OQ596209.1_Saussurea_superba	7	p2	(TA)5	10	18,257	18,266
OQ596209.1_Saussurea_superba	8	p2	(AT)5	10	19,253	19,262
OQ596209.1_Saussurea_superba	9	p6	(TATTCC)3	18	21,217	21,234
OQ596209.1_Saussurea_superba	10	p1	(T)12	12	22,851	22,862
OQ596209.1_Saussurea_superba	11	p1	(T)10	10	25,641	25,650
OQ596209.1_Saussurea_superba	12	p2	(AT)7	14	26,199	26,212
OQ596209.1_Saussurea_superba	13	p3	(TAA)4	12	30,673	30,684
OQ596209.1_Saussurea_superba	14	p5	(ACTAT)3	15	30,696	30,710
OQ596209.1_Saussurea_superba	15	p3	(TTC)4	12	34,146	34,157
OQ596209.1_Saussurea_superba	16	p1	(A)14	14	34,333	34,346
OQ596209.1_Saussurea_superba	17	p1	(T)10	10	34,352	34,361
OQ596209.1_Saussurea_superba	18	p1	(A)10	10	43,367	43,376
OQ596209.1_Saussurea_superba	19	p1	(A)15	15	43,621	43,635
OQ596209.1_Saussurea_superba	20	p1	(T)13	13	49,717	49,729
OQ596209.1_Saussurea_superba	21	p1	(T)13	13	54,071	54,083
OQ596209.1_Saussurea_superba	22	p6	(CCTGCT)3	18	58,250	58,267
OQ596209.1_Saussurea_superba	23	p3	(AAT)4	12	58,372	58,383
OQ596209.1_Saussurea_superba	24	p1	(T)10	10	58,717	58,726
OQ596209.1_Saussurea_superba	25	p1	(T)10	10	62,624	62,633
OQ596209.1_Saussurea_superba	26	p1	(T)17	17	64,448	64,464
OQ596209.1_Saussurea_superba	27	p1	(A)13	13	64,875	64,887
OQ596209.1_Saussurea_superba	28	p2	(TA)7	14	67,203	67,216
OQ596209.1_Saussurea_superba	29	p4	(TATT)3	12	67,261	67,272
OQ596209.1_Saussurea_superba	30	p1	(A)11	11	68,479	68,489
OQ596209.1_Saussurea_superba	31	p3	(ATA)4	12	70,549	70,560
OQ596209.1_Saussurea_superba	32	p1	(T)11	11	70,840	70,850
OQ596209.1_Saussurea_superba	33	p1	(A)12	12	77,189	77,200
OQ596209.1_Saussurea_superba	34	p1	(T)10	10	77,436	77,445
OQ596209.1_Saussurea_superba	35	p1	(T)16	16	79,868	79,883
OQ596209.1_Saussurea_superba	36	p1	(T)10	10	80,458	80,467
OQ596209.1_Saussurea_superba	37	p4	(TTTC)3	12	81,651	81,662
OQ596209.1_Saussurea_superba	38	p1	(A)12	12	106,942	106,953
OQ596209.1_Saussurea_superba	39	p1	(T)14	14	107,404	107,417
OQ596209.1_Saussurea_superba	40	p4	(AGAA)3	12	119,451	119,462
OQ596209.1_Saussurea_superba	41	p4	(AATC)3	12	119,858	119,869
OQ596209.1_Saussurea_superba	42	p4	(GATT)3	12	124,278	124,289
OQ596209.1_Saussurea_superba	43	p4	(CAAA)3	12	126,964	126,975
OQ596209.1_Saussurea_superba	44	p1	(A)13	13	128,535	128,547
OQ596209.1_Saussurea_superba	45	p1	(T)12	12	128,998	129,009

**Table 10:** SSRs in the chloroplast genome of *S. stella*.

ID	SSR nr.	SSR Type	SSR	Size	Start	End
OQ596210.1_Saussurea_stella	1	p1	(C)14	14	5334	5347
OQ596210.1_Saussurea_stella	2	p1	(A)11	11	8343	8353
OQ596210.1_Saussurea_stella	3	p1	(A)11	11	9444	9454
OQ596210.1_Saussurea_stella	4	p1	(A)10	10	13,013	13,022
OQ596210.1_Saussurea_stella	5	p1	(A)10	10	18,055	18,064
OQ596210.1_Saussurea_stella	6	p2	(TA)5	10	18,278	18,287
OQ596210.1_Saussurea_stella	7	p2	(AT)5	10	19,274	19,283
OQ596210.1_Saussurea_stella	8	p6	(TATTCC)3	18	21,238	21,255
OQ596210.1_Saussurea_stella	9	p1	(T)11	11	22,872	22,882
OQ596210.1_Saussurea_stella	10	p1	(T)10	10	25,673	25,682
OQ596210.1_Saussurea_stella	11	p2	(AT)6	12	26,231	26,242
OQ596210.1_Saussurea_stella	12	p3	(TAA)4	12	30,704	30,715
OQ596210.1_Saussurea_stella	13	p5	(ACTAT)3	15	30,727	30,741
OQ596210.1_Saussurea_stella	14	p3	(TTC)4	12	34,177	34,188
OQ596210.1_Saussurea_stella	15	p1	(A)10	10	34,364	34,373
OQ596210.1_Saussurea_stella	16	p1	(A)13	13	35,319	35,331
OQ596210.1_Saussurea_stella	17	p1	(A)18	18	43,681	43,698
OQ596210.1_Saussurea_stella	18	p1	(T)10	10	45,794	45,803
OQ596210.1_Saussurea_stella	19	p1	(T)10	10	46,158	46,167
OQ596210.1_Saussurea_stella	20	p1	(T)10	10	49,729	49,738
OQ596210.1_Saussurea_stella	21	p1	(T)14	14	54,058	54,071
OQ596210.1_Saussurea_stella	22	p3	(AAT)4	12	58,369	58,380
OQ596210.1_Saussurea_stella	23	p1	(T)11	11	58,721	58,731
OQ596210.1_Saussurea_stella	24	p1	(T)14	14	64,443	64,456
OQ596210.1_Saussurea_stella	25	p2	(TA)6	12	67,193	67,204
OQ596210.1_Saussurea_stella	26	p4	(TATT)3	12	67,249	67,260
OQ596210.1_Saussurea_stella	27	p3	(ATA)4	12	70,540	70,551
OQ596210.1_Saussurea_stella	28	p3	(AAT)4	12	74,675	74,686
OQ596210.1_Saussurea_stella	29	p1	(T)10	10	77,431	77,440
OQ596210.1_Saussurea_stella	30	p1	(T)14	14	79,863	79,876
OQ596210.1_Saussurea_stella	31	p1	(T)10	10	80,451	80,460
OQ596210.1_Saussurea_stella	32	p4	(TTTC)3	12	81,644	81,655
OQ596210.1_Saussurea_stella	33	p1	(T)10	10	81,682	81,691
OQ596210.1_Saussurea_stella	34	p1	(A)12	12	106,947	106,958
OQ596210.1_Saussurea_stella	35	p1	(T)13	13	107,409	107,421
OQ596210.1_Saussurea_stella	36	p4	(TTTA)3	12	111,628	111,639
OQ596210.1_Saussurea_stella	37	p4	(ATAA)3	12	117,553	117,564
OQ596210.1_Saussurea_stella	38	p4	(AGAA)3	12	119,398	119,409
OQ596210.1_Saussurea_stella	39	p4	(GATT)3	12	124,222	124,233
OQ596210.1_Saussurea_stella	40	p1	(T)12	12	124,837	124,848
OQ596210.1_Saussurea_stella	41	p4	(CAAA)3	12	126,908	126,919
OQ596210.1_Saussurea_stella	42	p1	(A)13	13	128,479	128,491
OQ596210.1_Saussurea_stella	43	p1	(T)12	12	128,942	128,953

**Table 11:** Codon usage bias analysis of the chloroplast genomes of *S. katochaete*, *S. superba*, and *S. stella*.

Codon	Amino Acid	*S. katochaete*			*S. superba*			*S. stella*		
		Quantity	Total	RSCU	Quantity	Total	RSCU	Quantity	Total	RSCU
UAA	*	29	54	1.61	29	52	1.67	29	54	1.61
UAG	*	13		0.72	12		0.69	14		0.78
UGA	*	12		0.67	11		0.63	11		0.61
GCU	A (Ala)	539	1210	1.78	494	1113	1.78	509	1154	1.76
GCC	A (Ala)	186		0.61	174		0.63	185		0.64
GCA	A (Ala)	349		1.15	313		1.12	328		1.14
GCG	A (Ala)	136		0.45	132		0.47	132		0.46
UGU	C (Cys)	166	230	1.44	141	189	1.49	148	203	1.46
UGC	C (Cys)	64		0.56	48		0.51	55		0.54
GAU	D (Asp)	688	861	1.6	560	710	1.58	574	725	1.58
GAC	D (Asp)	173		0.4	150		0.42	151		0.42
GAA	E (Glu)	837	1125	1.49	731	952	1.54	752	981	1.53
GAG	E (Glu)	288		0.51	221		0.46	229		0.47
UUU	F (Phe)	809	1204	1.34	702	988	1.42	707	1008	1.4
UUC	F (Phe)	395		0.66	286		0.58	301		0.6
GGU	G (Gly)	491	1497	1.31	456	1345	1.36	474	1410	1.34
GGC	G (Gly)	179		0.48	166		0.49	173		0.49
GGA	G (Gly)	572		1.53	500		1.49	523		1.48
GGG	G (Gly)	255		0.68	223		0.66	240		0.68
CAU	H (His)	396	510	1.55	347	446	1.56	354	452	1.57
CAC	H (His)	114		0.45	99		0.44	98		0.43
AUU	I (Ile)	896	1828	1.47	779	1561	1.5	811	1692	1.49
AUC	I (Ile)	350		0.57	284		0.55	302		0.56
AUA	I (Ile)	582		0.96	498		0.96	516		0.95
AAA	K (Lys)	855	1121	1.53	749	948	1.58	776	992	1.56
AAG	K (Lys)	266		0.47	199		0.42	216		0.44
UUA	L (Leu)	729	2246	1.95	662	1894	2.1	664	1942	2.05
UUG	L (Leu)	466		1.24	388		1.23	396		1.22
CUU	L (Leu)	481		1.28	394		1.25	406		1.25
CUC	L (Leu)	131		0.35	96		0.3	105		0.32
CUA	L (Leu)	299		0.8	246		0.78	257		0.79
CUG	L (Leu)	140		0.37	108		0.34	114		0.35
AUG	M (Met)	502	502	1	435	435	1	442	442	1
AAU	N (Asn)	807	1027	1.57	677	854	1.59	708	900	1.57
AAC	N (Asn)	220		0.43	177		0.41	192		0.43
CCU	P (Pro)	343	893	1.54	300	766	1.57	321	803	1.6
CCC	P (Pro)	159		0.71	134		0.7	140		0.7
CCA	P (Pro)	259		1.16	218		1.14	227		1.13
CCG	P (Pro)	132		0.59	114		0.6	115		0.57
CAA	Q (Gln)	592	766	1.55	513	649	1.58	529	677	1.56
CAG	Q (Gln)	174		0.45	136		0.42	148		0.44
CGU	R (Arg)	295	1274	1.39	280	1118	1.5	287	1148	1.5
CGC	R (Arg)	83		0.39	71		0.38	73		0.38
CGA	R (Arg)	284		1.34	250		1.34	262		1.37
CGG	R (Arg)	88		0.41	76		0.41	74		0.39
AGA	R (Arg)	385		1.81	334		1.79	344		1.8
AGG	R (Arg)	139		0.65	107		0.57	108		0.56
UCU	S (Ser)	470	1598	1.76	393	1293	1.82	398	1335	1.79
UCC	S (Ser)	244		0.92	181		0.84	196		0.88
UCA	S (Ser)	319		1.2	256		1.19	259		1.16
UCG	S (Ser)	138		0.52	102		0.47	108		0.49
AGU	S (Ser)	333		1.25	290		1.35	294		1.32
AGC	S (Ser)	94		0.35	71		0.33	80		0.36
ACU	T (Thr)	439	1072	1.64	388	942	1.65	400	976	1.64
ACC	T (Thr)	203		0.76	185		0.79	195		0.8
ACA	T (Thr)	335		1.25	296		1.26	304		1.25
ACG	T (Thr)	95		0.35	73		0.31	77		0.32
GUU	V (Val)	422	1170	1.44	378	1040	1.45	386	1083	1.43
GUC	V (Val)	144		0.49	124		0.48	129		0.48
GUA	V (Val)	447		1.53	407		1.57	428		1.58
GUG	V (Val)	157		0.54	131		0.5	140		0.52
UGG	W (Trp)	387	387	1	334	334	1	337	337	1
UAU	Y (Tyr)	671	833	1.61	574	712	1.61	589	735	1.6
UAC	Y (Tyr)	162		0.39	138		0.39	146		0.4
			21,408			18,341			18,986	

“*” denotes a termination codon.

**Table 12:** Plant samples used in this study.

Species	Subtribe	Tribe	Origin	Accession Number
*C. tinctorius*	Centaureinae	Trib. Cynareae	NCBI	NC030783.1
*C. cyanus*			NCBI	NC066898.1
*A. lancea*	Carlininae		NCBI	MT834522.1
*C. acaulis*			NCBI	NC063929.1
*A. lappa*	Carduinae		NCBI	MH375874.1
*C. crispus*			NCBI	MK652229.1
*C. japonicum*			NCBI	NC053767.1
*C. cardunculus var. scolymus*			NCBI	KM035764.1
*D. calophylla*			NCBI	MT128668.1
***S. katochaete***			Qinghai, China	OQ596208.1
***S. superba***			Qinghai, China	OQ596209.1
***S. stella***			Qinghai, China	OQ596210.1
*S. salicifolia*			NCBI	NC057621.1
*S. medusa*			NCBI	MZ128902.1
*S. sutchuenensis*			NCBI	MK966029.1
*S. apus*			NCBI	MH926074.1
*S. depsangensis*			NCBI	MH926091.1
*S. gossipiphora*			NCBI	MH926100.1
*S. phaeantha*			NCBI	MT554930.1
*S. inversa*			NCBI	NC050977.1
*S. albifolia*			NCBI	MT478053.1
*S. salsa*			NCBI	MH926157
*S. oligocephala*			NCBI	NC067031.1
*S. brachycephala*			NCBI	MH926079.1
*S. amurensis*			NCBI	MH926072.1
*S. bullockii*			NCBI	MK966027.1
*S. leucophylla*			NCBI	MH926118.1
*S. tomentosa*			NCBI	MH926177.1
*S. komaroviana*			NCBI	MH926112.1
*H. annuus*	/	Trib. Heliantheae	NCBI	NC007977.1

The three species included in this study are bolded.

## Data Availability

The assembled chloroplast genome sequences of *S. katochaete*, *S. superba,* and *S. stella* have been uploaded to and deposited in GenBank under accession numbers OQ596208, OQ596209, and OQ596210, respectively. The first author and corresponding author can provide all relevant raw data if required.

## Supplementary Materials

The following supporting information can be downloaded at: https://www.mdpi.com/article/10.3390/genes14112002/s1, Figure S1: Comparison of the genomes of Saussurea species using Geneious Prime. Annotations of the CDs, rRNA, and tRNA genes are shown in yellow, red, and with purple-red arrowheads, respectively.
