# Supplementary material for: Complete Chloroplast Genomes of Saussurea katochaete, Saussurea superba, and Saussurea stella: Genome Structures and Comparative and Phylogenetic Analyses
Source: Genes (Basel). 2023 Oct 26;14(11):2002. doi: 10.3390/genes14112002 (PMC10670953; doi:10.3390/genes14112002)
Supplement: Supplementary file 1 [file genes-14-02002-s001.zip › genes-2646117-supplementary.pdf]

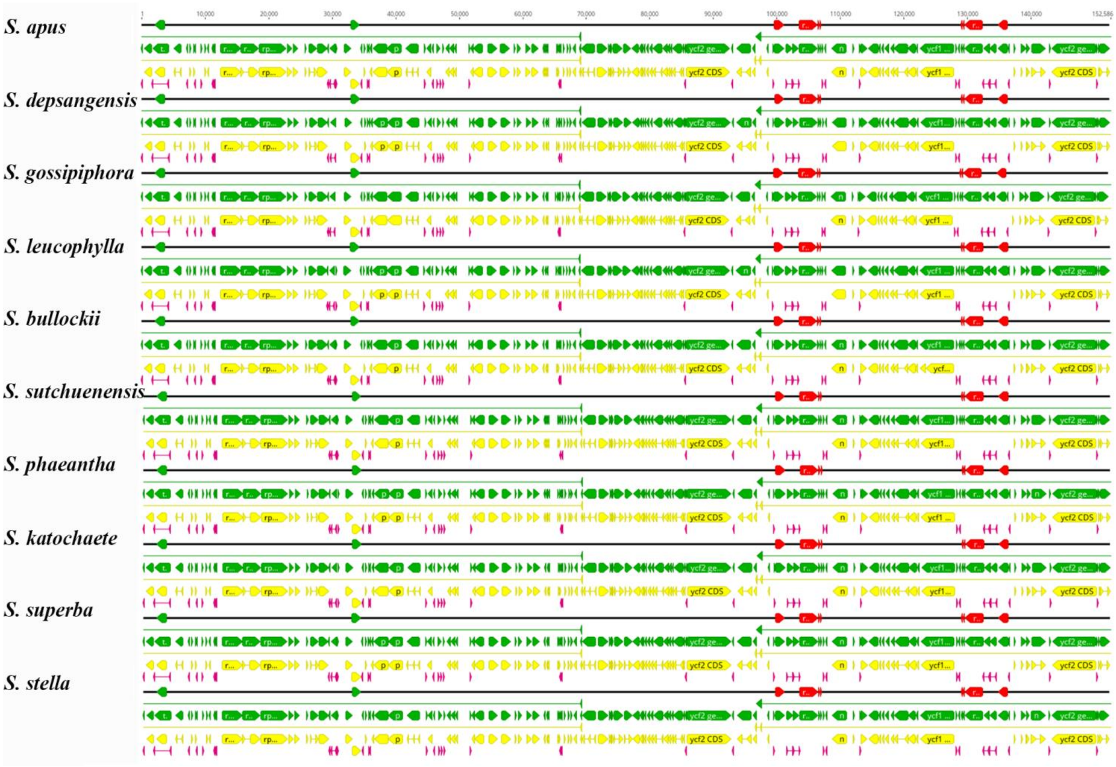

Supplementary Figure S1: Comparison of the genomes of *Saussurea* species using Geneious Prime. Annotations of the CDs, rRNA, and tRNA genes are shown in yellow, red, and with purple-red arrowheads, respectively.
